# Class I Histone Deacetylase Inhibitor Entinostat Suppresses Regulatory T Cells and Enhances Immunotherapies in Renal and Prostate Cancer Models

**DOI:** 10.1371/journal.pone.0030815

**Published:** 2012-01-27

**Authors:** Li Shen, Michael Ciesielski, Swathi Ramakrishnan, Kiersten M. Miles, Leigh Ellis, Paula Sotomayor, Protul Shrikant, Robert Fenstermaker, Roberto Pili

**Affiliations:** 1 Department of Medicine, Roswell Park Cancer Institute, Buffalo, New York, United States of America; 2 Department of Neuro-Oncology, Roswell Park Cancer Institute, Buffalo, New York, United States of America; 3 Department of Immunology, Roswell Park Cancer Institute, Buffalo, New York, United States of America; Queensland University of Technology, Australia

## Abstract

**Background:**

Immunosuppressive factors such as regulatory T cells (Tregs) limit the efficacy of immunotherapies. Histone deacetylase (HDAC) inhibitors have been reported to have antitumor activity in different malignancies and immunomodulatory effects. Herein, we report the Tregs-targeting and immune-promoting effect of a class I specific HDAC inhibitor, entinostat, in combination with either IL-2 in a murine renal cell carcinoma (RENCA) model or a survivin-based vaccine therapy (SurVaxM) in a castration resistant prostate cancer (CR Myc-CaP) model.

**Methods and Results:**

RENCA or CR Myc-CaP tumors were implanted orthotopically or subcutaneously, respectively. Inoculated mice were randomized into four treatment groups: vehicle, entinostat, cytokine or vaccine, and combination. Tregs in the blood were assessed by FACS analysis. Real time quantitative PCR and Western blot analysis of isolated T cell subpopulations from spleen were performed to determine Foxp3 gene and protein expression. The suppressive function of Tregs was tested by T cell proliferation assay. Low dose (5 mg/kg) entinostat reduced Foxp3 levels in Tregs and this was associated with enhanced tumor growth inhibition in combination with either IL-2 or a SurVaxM vaccine. Entinostat down-regulated Foxp3 expression transcriptionally and blocked Tregs suppressive function without affecting T effector cells (Teffs). *In vitro* low dose entinostat (0.5 µM) induced STAT3 acetylation and a specific inhibitor of STAT3 partially rescued entinostat-induced down-regulation of Foxp3, suggesting that STAT3 signaling is involved in Foxp3 down-regulation by entinostat.

**Conclusions:**

These results demonstrate a novel immunomodulatory effect of class I HDAC inhibition and provide a rationale for the clinical testing of entinostat to enhance cancer immunotherapy.

## Introduction

Tumor growth represents an outcome of tumor cells escaping host immune surveillance. Despite some successes, immunotherapeutic interventions have shown limited benefit. A major barrier is represented by the presence of immunosuppressive factors that appear to be predominant in cancer patients. These immunosuppressive components include Tregs [1], [2], myeloid derived suppressor cells (MDSCs), immunological checkpoints mediated by cell surface molecules such as CTLA-4 [3] and PD-1 [4], and circulating cytokines such as TGF-β and IL-10 [5]. Studies have shown that these tolerance mechanisms can be induced by tumor and surrounding stromal cells. Tregs normally maintain the tolerance for self-antigens and prevent autoimmune responses [6], [7]. On the other hand, Tregs have been identified as one of the major players in tumor immune tolerance. The supporting evidence includes Tregs promotion in cancer patients and Tregs expansion following immunotherapy [2], [8]–[11]. Further clinical reports suggest that depletion of Tregs may enhance an antitumor immune response in cancer patients.

High dose IL-2 is an FDA-approved treatment for selected patients with metastatic clear cell renal cell cancer [12], [13]. IL-2 therapy induces objective responses in about 20% of patients, with durable complete responses in a small fraction. Given the limited efficacy of high dose IL-2 therapy, additional efforts have been directed to increase the efficacy of this immunotherapeutic approach.

Vaccine therapies remain of limited benefit in solid tumors, though the vaccine therapy Sipuleucel-T was recently approved for the treatment of castration resistant prostate cancer. Tregs are predominant in various cancers, including advanced prostate cancer [2]. Studies have shown that the presence of immunosuppressive factors such as Tregs play an important role in immune tolerance and low efficacy in vaccine therapy [14], [15]. Accordingly, combination of vaccines with approach(es) to deplete or suppress Tregs represents a rational strategy in prostate cancer therapy.

HDACs have been shown to be involved in oncogenic transformation by mediating the transcriptional regulation of genes that are involved in cell cycle progression, proliferation, and apoptosis [16], [17]. HDAC inhibitors are currently being developed for cancer treatment and have demonstrated antitumor activity in different tumors. HDACs have been characterized into four different classes with different targets and subcellular locations. In addition to histones, several non-histone proteins are also reversibly acetylated at lysine residues and these post-translational modifications may also play an important role in the antitumor effects of HDAC inhibitors [18]–[20]. The synthetic benzamide, entinostat, is a selective inhibitor of class I HDACs. Entinostat has antitumor activity both *in vitro* and *in vivo* in several tumor models [21]–[24]. In addition, our group has previously reported the synergistic antitumor activity of entinostat in combination with high dose IL-2 in the RENCA model [25].

Recent experimental studies have demonstrated that HDAC inhibitors have potential immunomodulatory activity in both i*n vitro* and *in vivo* models of inflammation, autoimmunity, and transplantation. HDAC inhibitors can affect immune responses by regulating the production of cytokines. In a murine model of allogeneic bone marrow transplantation, the HDAC inhibitor, vorinostat (SAHA), reduced acute graft-versus-host disease by suppression of pro-inflammation cytokines such as TNF-α, IL-1, and INF-γ [26]. The HDAC inhibitor, LAQ824, has been shown to alter activation and function of macrophage and dendritic cells. LAQ824 has also been found to modulate dendritic cell function to inhibit Th1, but not Th2 effector function [27]. In addition, HDAC inhibitors can regulate the transcription of major histocompatibility class I and II [28], or the activation of co-stimulatory molecules [28], [29]. More recently, it has been reported that a pan HDAC inhibitor, tricostatin A (TSA) may increase the function of Tregs and enhance their immunosuppressive effect *in vivo* [30]. Moreover, the positive results from clinical trials in cutaneous T cell lymphomas (CTCL) suggest that HDAC inhibitors may affect the immune response, since some of the pathological mechanisms of CTCL are mediated through inflammation and an imbalance of the immune system. Taken together, these observations suggest that the antitumor activity of HDAC inhibitors may be in part due to their immunomodulatory properties.

In this study, we have different results in our system and report that treatment with entinostat decreases Foxp3 expression in Tregs and inhibits the suppressive function of Tregs. In addition, STAT3 signaling was shown to be associated with Foxp3 down-regulation by entinostat. This property of entinostat may enhance the antitumor immune response to IL-2 and vaccine therapy and provides a rationale for using entinostat in combination strategies with immunotherapies.

## Results

### Entinostat enhancing high dose IL-2 therapy is associated with modulation of Tregs in tumor-bearing mice

**Figure 1:**
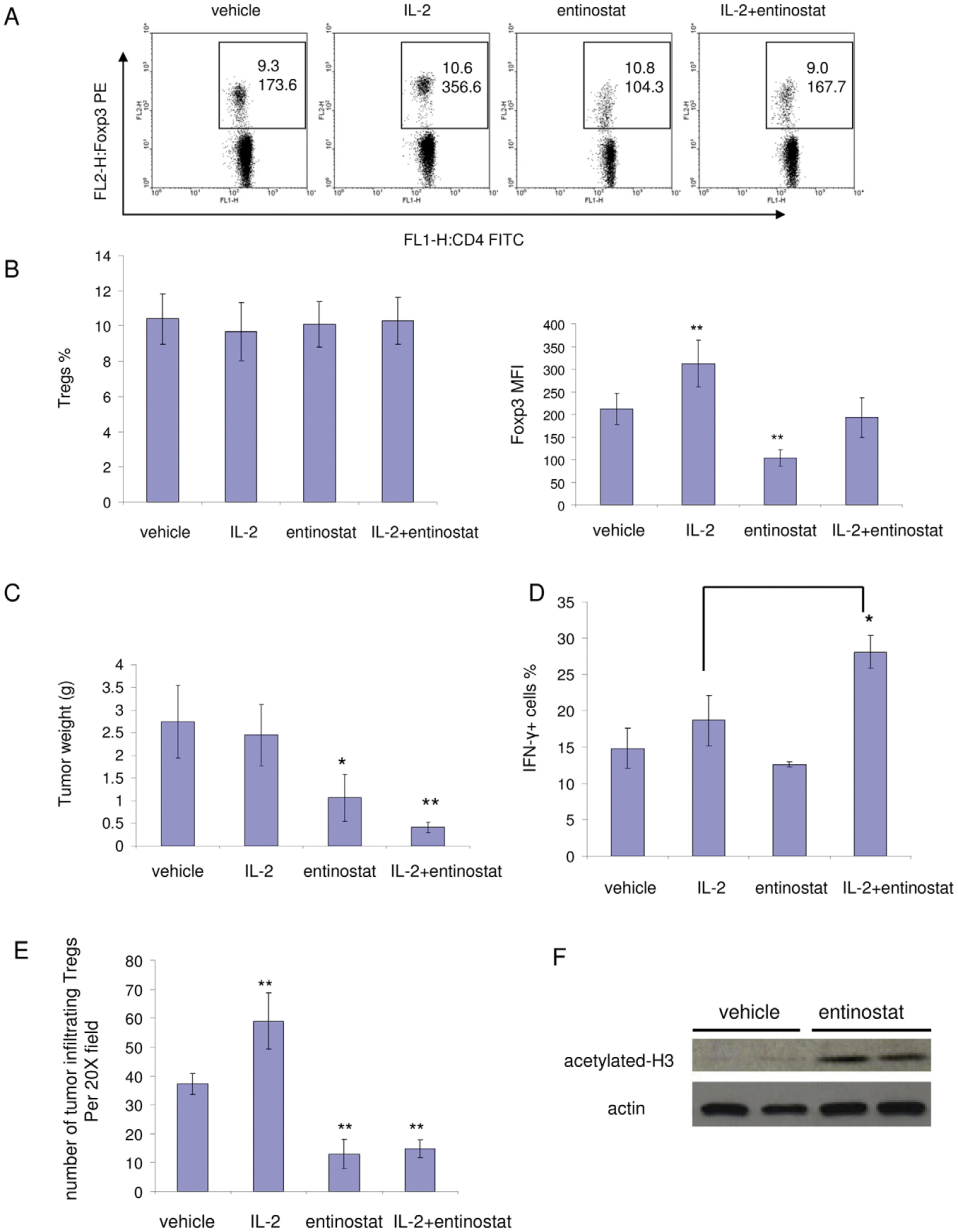
Enhancement of IL-2 therapy by entinostat is associated with inhibition of Tregs. Mice with orthotopic inoculation of RENCA cells were treated for 5 days. Blood was drawn from mice on the fifth day and stained for antibodies specific for CD4, CD25, and Foxp3. Tumor weights were measured at the end of two weeks of treatment. A and B, Effect of entinostat on Tregs in tumor bearing mice. A, Effects of vehicle, IL-2, entinostat, or combination treatment on Tregs Foxp3 expression. Cells were stained and subjected to flow cytometry analysis. The dot plots were gated for CD4^+^ cells. The rectangular area encloses the CD4^+^Foxp3^+^ cells, the numbers on the top represent the percentage of Foxp3^+^ cells. The numbers on the bottom in the area represent the mean fluorescence intensity (MFI) of Foxp3 PE staining of CD4^+^Foxp3^+^ cells. B, Quantification of Tregs percentage in CD4 population (left panel) and Foxp3 levels (MFI) in Tregs (right panel) by FACS analysis. Values are means and error bars represent S.D. for 5–7 samples per group. In right panel, *p* = 0.0011 for IL-2 vs. vehicle; *p* = 0.000009 for entinostat vs. vehicle. Results are representative of three separate experiments. C, Tumor weight measurements. Columns, mean grams of tumor; Bars, S.D. n = 5–7. *p* = 0.0209 for entinostat vs. vehicle; *p* = 0.0077 for combination vs. vehicle; *p* = 0.0272 for combination vs. entinostat. Results are representative of three separate experiments. D, Entinostat enhanced IFN-γ type immune response induced by IL-2 treatment. Splenocytes (1×10^6^ cells) were harvested and stimulated with PMA (20 ng/ml) and Ionomycin (1 µg/ml) for 5 hours in the presence of Brefeldin A. Cells were then stained for surface markers and intracellular IFN-γ. Histograms show percentage of IFN-γ expressing cells in CD8 population. *p* = 0.01 for combination vs. IL-2. E, Entinostat reduced tumor infiltration of Tregs. Tumor sections were stained with anti-Foxp3 antibody to show infiltration of Tregs. Histogram shows average numbers of stained Tregs in random 20× resolution bright fields (Tregs number in each field was obtained by blinded count). F, Entinostat treatment induced H3 histone acetylation in splenocytes. BALB/c mice were treated with vehicle (0.5% methocel) or 5 mg/kg/day entinostat by gavage for 5 days. Cells were harvested from spleens and subjected to Western blot analysis for acetylated-H3 histone.

Our group previously reported that the class I HDAC inhibitor, entinostat, has an antitumor effect in the RENCA model [25], [31]. Entinostat appears to have an immunomodulatory effect that leads to a synergistic antitumor effect in combination with IL-2. IL-2 treatment promotes proliferation and activation of T effector cells (Teffs), but also induces immunosuppressive Tregs with stable expression of the IL-2 receptor CD25. Therefore, in the current study, we focused on the effect of entinostat on Tregs. We tested the effect of entinostat as a single agent and in combination with IL-2 on Tregs in the RENCA model. RENCA cells were inoculated orthotopically in BALB/c mice. Three days after inoculation, animals received treatment with either vehicle, IL-2, entinostat (5 mg/kg), or combination. After 5 days of treatment, peripheral blood was collected from each mouse, stained for cell surface markers and intracellular Foxp3 protein, and subjected to fluorescence associated cell sorting (FACS) analysis. No significant differences were observed in the numbers of CD4^+^Foxp3^+^ Tregs (Fig. 1A and B). However, Foxp3 protein levels in CD4^+^Foxp3^+^ cells, as represented as mean fluorescence intensity (MFI), decreased with entinostat treatment (Fig. 1A and B). An increase in Foxp3 levels was observed with IL-2 treatment alone, confirming the notion that IL-2 promotes Tregs while supporting T cell proliferation. In combination treatment, entinostat still rescued Foxp3 levels back to control levels (Fig. 1A and B). Western blot analyses showed that *in vivo* entinostat treatment increased the acetylation level of H3 histone in splenocytes (Fig. 1F). Antitumor effects of treatments were evaluated by assessing tumor weights after two weeks. No significant body weight changes were observed with treatments. IL-2 treatment induced a modest reduction of tumor weight (<10%). Entinostat single agent administration at a 5 mg/kg led to a significant tumor weight reduction as compared to control group (∼40% reduction, Fig. 1C). The combination of entinostat with IL-2 had a much greater inhibitory effect on tumor growth (∼80% reduction, Fig. 1C). To determine whether the reduction of Foxp3 expression and inhibition of tumor growth induced by entinostat was associated with increased immune response, we examined IFN-γ induction. IFN-γ was slightly induced in CD8 cells in IL-2-treated animals, while CD8 cells in combination-treated animals had much higher IFN-γ induction (Fig. 1D). Taken together, these observations suggest that entinostat enhances CD8 cell immune response induced by IL-2 while reducing Foxp3 level in Tregs.

We examined whether entinostat treatment affected the Tregs that were infiltrating the tumors. Immunohistochemistry staining of the tumor sections demonstrated that the entinostat treatment reduced Tregs infiltration (Fig. 1E and Fig. S1).

**Figure 2:**
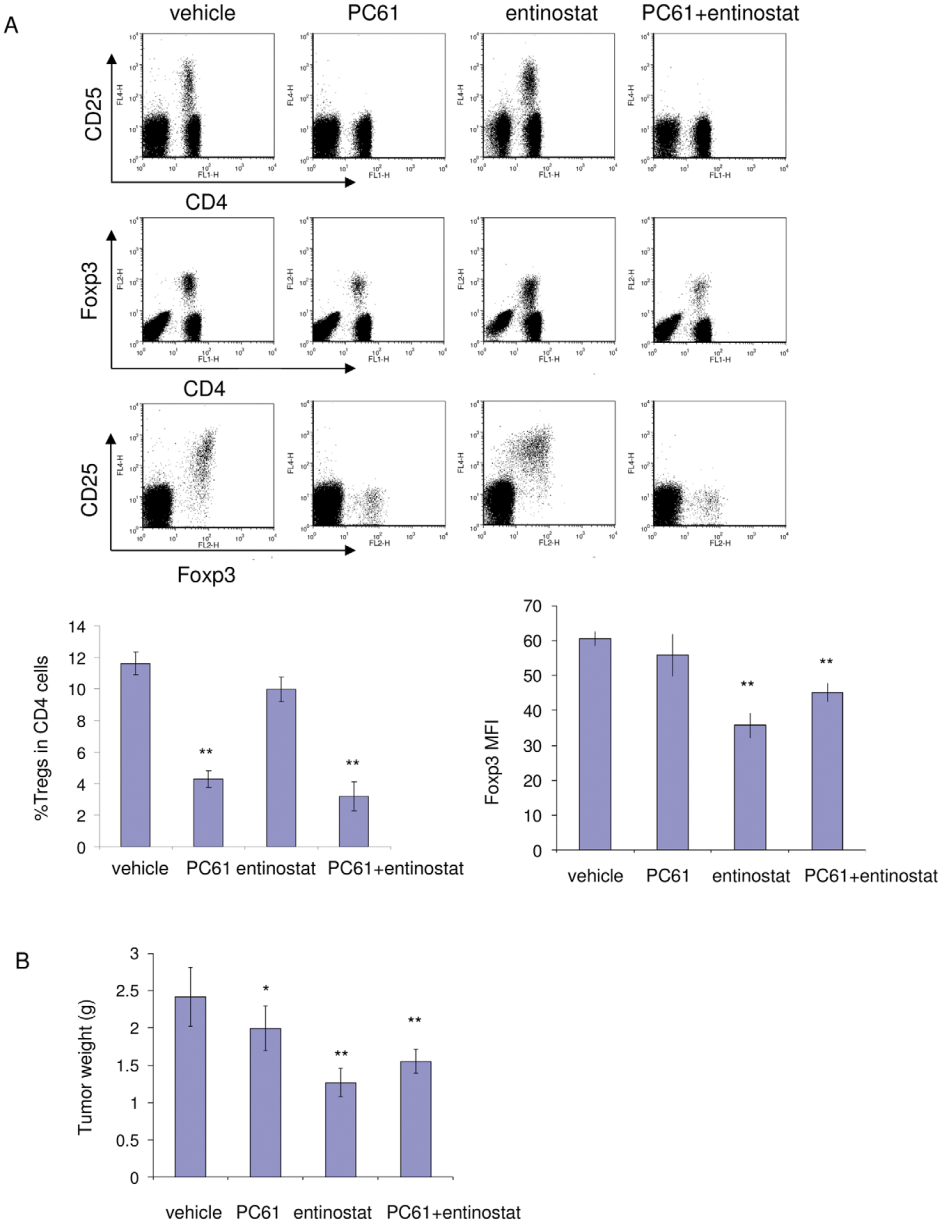
Antitumor activity of Tregs depletion antibody PC61 in RENCA tumor. A, Plots show effect of vehicle, PC61, entinostat or combination treatment on CD25 and Foxp3 levels. Quantification of Tregs percentage, Foxp3 expression by FACS analysis is shown as histograms. Values are means and error bars represent S.D. for 6–7 samples per group. B, Tumor weight measurements from PC61 depletion experiment. Columns, mean grams of tumor; Bars, S.D.. n = 6–7. * represents *p*<0.05 for marked point vs. vehicle; ** represents *p*<0.01 for marked point vs. vehicle.

We also tested the anti-mouse CD25 antibody, PC61, to deplete Tregs [32] in the RENCA model. PC61 treatment at 500 µg/mouse/wk was sufficient to deplete CD25^+^ cells (Fig. 2A), dramatically reduced CD4^+^Foxp3^+^ Tregs cell number (Fig. 2A), and had a similar antitumor effect as observed with entinostat (Fig. 2B). In addition, adding PC61 treatment did not have an additional antitumor activity over entinostat treatment (Fig. 2B), which suggests that entinostat and PC61 may have a redundant mechanism of activity.

### Entinostat synergizes with peptide vaccine therapy in a castration resistant prostate cancer model

**Figure 3:**
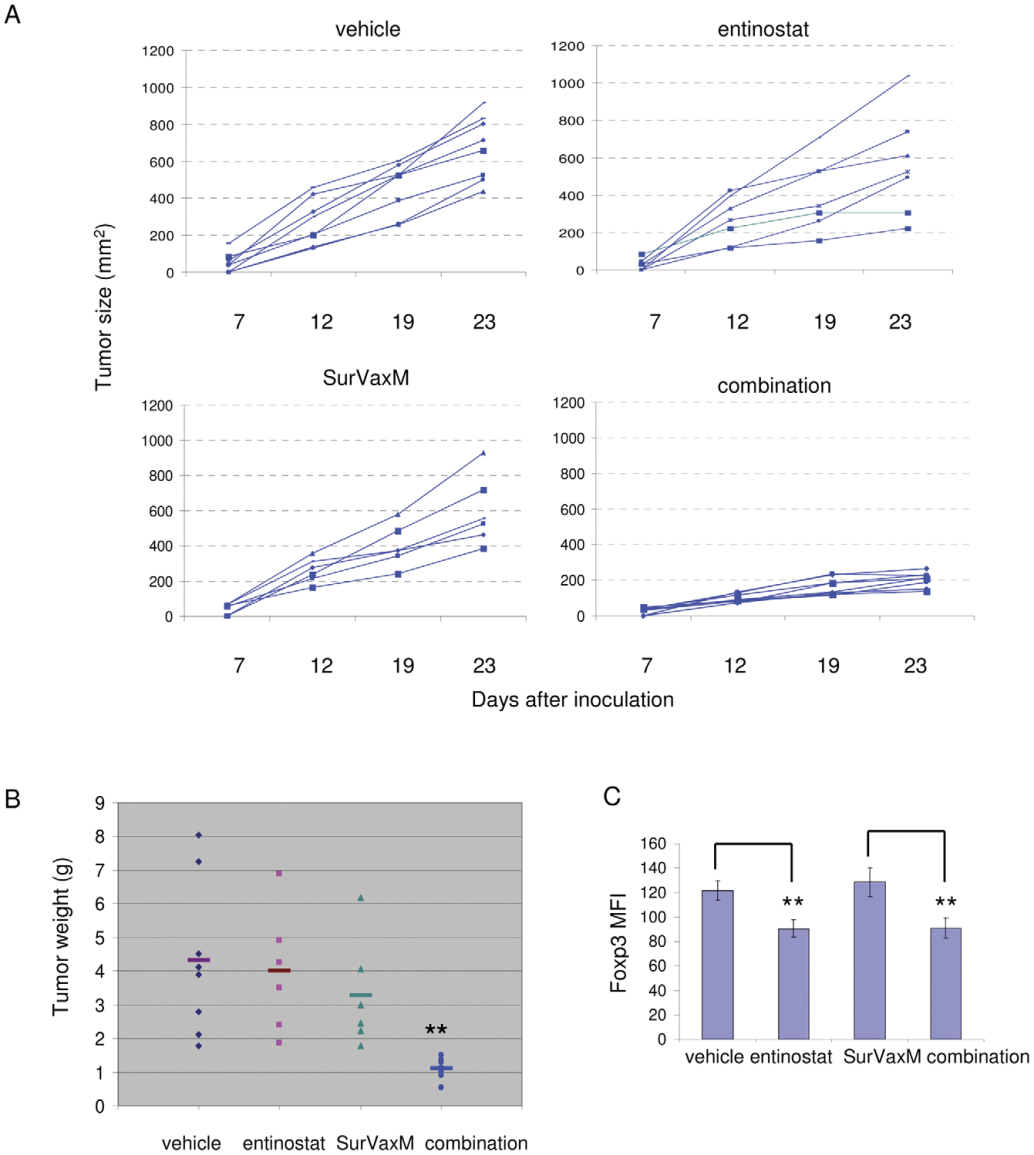
The HDAC inhibitor, entinostat, enhances peptide vaccine therapy in a castration resistant prostate cancer model. A and B, Entinostat enhances the antitumor effect of the survivin vaccine, SurVaxM. FVB mice were castrated and subcutaneously inoculated with small castration resistant tumor pieces. Approximately 7 days after inoculation when tumors reached an average size of 50 mm^2^, mice were treated three weeks with vehicle, entinostat (5 mg/kg, 1 dose/day, 5 days/wk), survivin vaccine SurVaxM (100 µg/dose, 1 dose/wk), or combination. A, Single tumor growth graph lines were generated by serial caliper measurements. B, Tumors were harvested at the end of treatment and weighed (combination vs. survivin vaccine, *p* = 0.003). C, Entinostat treatment reduced Foxp3 levels in Tregs from CR Myc-CaP tumor-bearing mice. After 5 days of treatment, peripheral blood cells were collected from mice, stained for CD4 and Foxp3, and subjected to FACS analysis. Quantitation of Foxp3 mean fluorescence intensity (Foxp3 MFI) (vehicle vs. entinostat, *p* = 0.0002; combination vs. survivin, *p* = 0.0004).

In addition to a cytokine therapy, we also tested entinostat in combination with another immunotherapeutic approach, a peptide vaccine therapy, in a castration resistant prostate cancer (CR Myc-CaP) model. We used a novel modified survivin peptide vaccine SVN53-67/M57-KLH (SurVaxM) [33]. Survivin is an intracellular tumor-associated antigen expressed in solid tumors, including prostate cancer. The level of survivin expression is associated with tumor progression and aggressiveness [34], and represents a suitable target for vaccine therapy. A transplantable castration resistant prostate cancer (CR Myc-CaP) model has been developed in our lab [35]. Myc-CaP cells, derived from the Hi-Myc transgenic prostate cancer mouse model [36], were injected (1×10^6^ cells/mouse) subcutaneously into male FVB mice. Tumor bearing animals were surgically castrated post tumor establishment and consequent tumors were passaged through 5 additional rounds of surgically castrated FVB mice. Survivin expression was confirmed in Myc-CaP tumors by immunohistochemistry. CR Myc-CaP tumor bearing mice were randomized into four groups and treated with vehicle, entinostat (5 days/wk, 5 mg/kg), SurVaxM (1 dose/wk) or combination. Following three weeks of treatment, entinostat or SurVaxM single treatment displayed modest antitumor effect (10–25% of reduction) (Fig. 3A and B). However, combination of entinostat and SurVaxM dramatically reduced tumor weight (∼80% reduction, *p* = 0.002) when compared to either vehicle or single treatment groups (Fig. 3A and B). Peripheral blood cell staining showed that treatment with entinostat alone and in combination with SurVaxM reduced Foxp3 level in Tregs of tumor-bearing mice (Fig. 3C), but had no effect on Tregs number (data not shown).

### Survivin vaccine treatment induces antigen-specific CD8 cells and entinostat synergizes with vaccine to induce IFN-γ immune response

**Figure 4:**
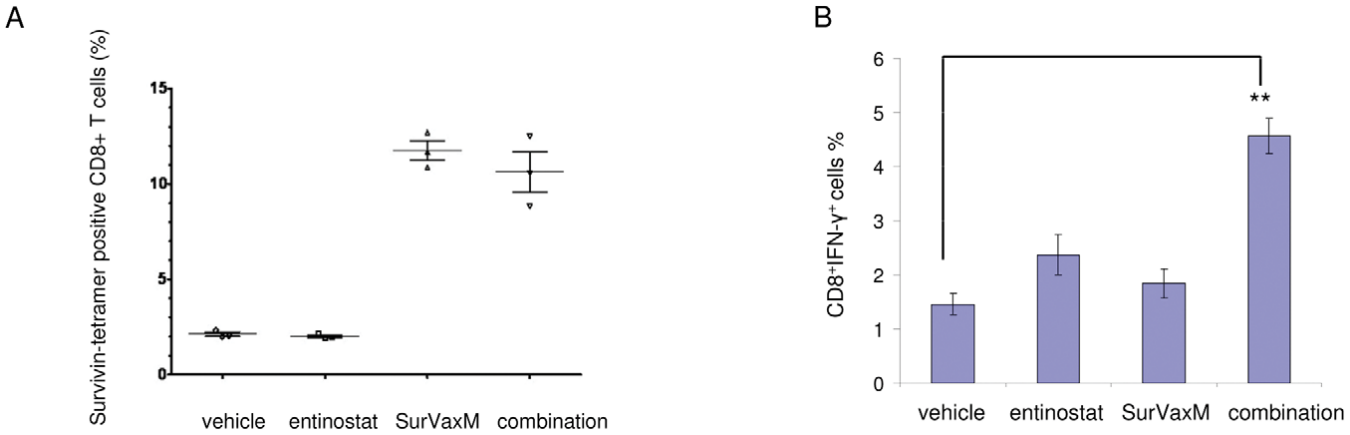
Survivin vaccine induces antigen-specific CD8 cells and entinostat enhances IFN-γ induction. A. Tetramer analysis of splenocytes obtained from mice immunized with SurVaxM. Splenocytes were stained with anti CD8 antibodies and survivin-specific tetramers for flow cytometric analysis. Results are based upon gating of CD8+ T cells and indicate the percent of double labeled cells (CD8+/Tetramer+) with respect to specific tetramer. B, Combination treatment led to CD8^+^ IFN-γ^+^ cells induction. Mice were treated as indicated. Splenocytes were stimulated and intracellular IFN-γ staining was performed as described in Figure 1D. *p*<0.01 for combination vs. vehicle.

In order to assess the presence of survivin antigen-specific T cells that may be generated in response to vaccination, we did a survivin vaccine-specific peptide-MHC class I tetramer binding assay. The tetramer is specific to a survivin peptide epitope [33]. Splenocytes were isolated from mice that received different treatments as done in the therapy experiment and subjected to survivin–specific tetramer and surface marker staining. Only the splenocytes from mice treated with the survivin vaccine (vaccine single and combination treatment) showed induction of antigen-specific CD8 cells (Fig. 4A). Interestingly, the intracellular cytokine staining suggests substantial induction of CD8^+^IFN-γ^+^ cells over vehicle level in the combination group, which matches its enhanced antitumor activity (Fig. 4B).

### Entinostat suppresses Foxp3 gene expression in Treg cells and inhibits Tregs function

**Figure 5:**
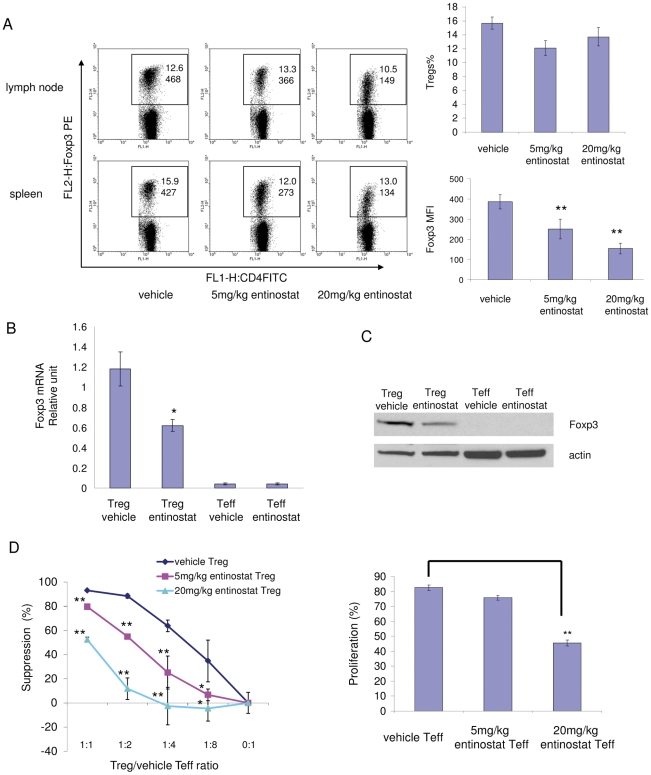
*In vivo* treatment with entinostat decreases Foxp3 expression in Tregs and inhibits Tregs function. BALB/c mice were treated with vehicle (0.5% methocel) or 5 mg/kg/day or 20 mg/kg/day of entinostat by gavage for 5 days. Cells were harvested from spleens and lymph nodes. A, *In vivo* entinostat treatment decreased the expression level of Foxp3 in CD4^+^Foxp3^+^ (Treg) cells. Cells were stained with fluorescence-conjugated antibodies specific to CD4, CD25, and Foxp3 and subjected to flow cytometry analysis. The dot plots were gated for CD4^+^ cells. The rectangular area encloses the CD4^+^Foxp3^+^ cells, the numbers on the top represent the percentage of Foxp3^+^ cells. The numbers on the bottom in the area represent the mean fluorescence intensity of Foxp3 PE staining of CD4^+^Foxp3^+^ cells. The panels on the right represent quantification of Tregs percentage (top) and Foxp3 expression (bottom) in FACS analysis. Graph shows means, error bars represent standard deviations for 6 samples in each group. *p* = 0.00078 for 5 mg/kg entinostat vs. vehicle; *p* = 0.000004 for 20 mg/kg entinostat vs. vehicle; *p* = 0.0038 for 20 mg/kg entinostat vs. 5 mg/kg entinostat. α = 0.05. Results are representative of three separate experiments. B, *In vivo* entinostat treatment reduced Foxp3 mRNA levels in isolated Tregs. Total RNAs were extracted from isolated CD4^+^CD25^+^ T cells (Tregs) and CD4^+^CD25^−^ T cells (Teffs) and were analyzed for Foxp3 mRNA by real time RT-PCR. Foxp3 mRNA levels were normalized to GAPDH mRNA (or ribosomal RNA RPL13A) and values represent means for three samples (three mice per sample) per group and have relative units. *p* = 0.0157 for Tregs treated with entinostat vs. Tregs treated with vehicle. Results are representative of three separate experiments. C, *In vivo* entinostat treatment reduced Foxp3 protein level in Tregs. Total cell protein was extracted from isolated Tregs and Teffs and was analyzed for Foxp3 protein by Western blot. D, The effects of HDAC inhibitor, entinostat, on Tregs suppressive function and Teffs proliferation. Tregs suppression assays: 2×10^5^ CFSE-labeled Teffs were stimulated with 0.5 µg/ml of anti-CD3ε antibody and 4×10^5^ irradiated antigen presenting cells (CD4 cells-depleted splenocytes) and co-cultured with Tregs in different ratios to Teffs for 62–80 hrs. Percentages of divided Teffs were calculated at all ratio points for each treatment. Percentage of suppression of Teffs dividing was calculated for each point by comparing cell dividing at each ratio to the cell dividing in absence of Tregs (0∶1). Left panel: Effect of entinostat treatment on Tregs function. Right panel: Effect of entinostat treatment on proliferation of Teffs. Histogram shows percentage of divided Teffs without Tregs. Data are representative of three separate experiments. Values are means from triplicate measurements. * represents *p*<0.05 for marked point vs. vehicle; ** represents *p*<0.01 for marked point vs. vehicle.

To further investigate the immune promoting effect of entinostat, we treated naïve BALB/c mice with either vehicle or entinostat (5 mg/kg or 20 mg/kg) for 5 days. Splenocytes and lymph node cells were harvested. The number of Tregs and Foxp3 expression were accessed by FACS analysis. *In vivo* treatment with entinostat had no significant effect on the number of Tregs (CD4^+^Foxp3^+^ T cells) in CD4^+^ T cell population from either lymph nodes or spleen. However, compared to vehicle-treated mice, Tregs from treated mice had a dose-dependent decrease in Foxp3 levels (Fig. 5A). The effect of entinostat on Foxp3 expression was also tested by measuring Foxp3 mRNA levels in isolated cell populations by quantitative real time RT-PCR. Tregs (CD4^+^CD25^+^) and non-Tregs (CD4^+^CD25^−^) CD4 T cells were purified from entinostat- and vehicle- treated mice by using magnetic beads. *In vivo* entinostat treatment significantly decreased Foxp3 messenger RNA in Tregs, as compared to Tregs from vehicle treated mice (Fig. 5B). The reduced Foxp3 protein expression in treated Tregs was also confirmed by Western blot analysis (Fig. 5C).

To determine whether decreased Foxp3 expression in entinostat treated Tregs leads to impaired suppressive function of Tregs, CFSE-labeled purified CD4^+^CD25^−^ T cells (Teffs) were cultured with anti-CD3e antibody and antigen presenting cells. Tregs were then added into the culture with different Treg vs. Teff ratios (Fig. 5D). BALB/c mice were treated with vehicle or different doses of entinostat *in vivo* as indicated. Tregs were isolated from splenocytes from differentially treated mice and cultured with isolated Teffs from vehicle-treated mice to test the effect of treatment on Tregs suppressive activities (Fig. 5D, left panel). In addition, Teffs isolated from splenocytes from differentially treated mice were stimulated to test the effects of different treatments on proliferation capacity of Teffs (Fig. 5D, right panel). Entinostat (5 mg/kg) treated Tregs were two to three times (for example, at Tregs∶Teff = 1∶4, 25% vs. 63% suppression) less effective in suppressing Teffs proliferation than vehicle-treated Tregs (Fig. 5D, left panel). Higher entinostat dose (20 mg/kg) further inhibited Treg suppressive function with up to a seven fold (Tregs∶Teffs = 1∶2) reduction (Fig. 5D, left panel). Interestingly, *in vivo* low dose entinostat treatment (5 mg/kg) showed minimal inhibition of proliferation capacity of Teffs, whereas higher dose (20 mg/kg) significantly inhibited the proliferation capacity of Teffs, as compared to vehicle-treated Teffs (Fig. 5D, right panel). Taken together, these results suggest that *in vivo* treatment with low, not cytotoxic dose of entinostat [25] inhibits Tregs suppressive function with minimal influence on Teffs proliferating capacity.

### STAT3 is acetylated by entinostat treatment and is associated with Foxp3 down-regulation

**Figure 6:**
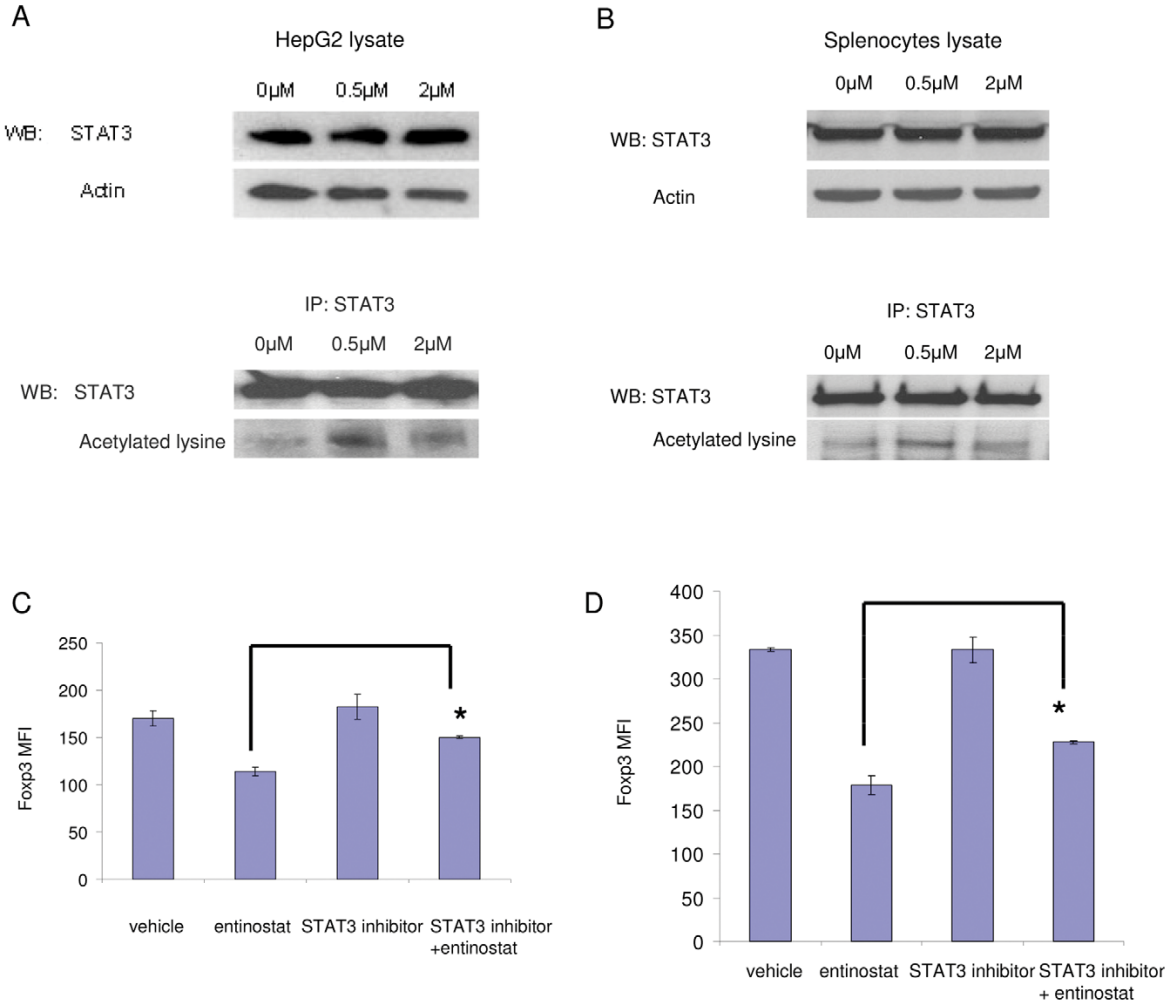
STAT3 signaling is involved in down-regulation of Foxp3 by entinostat. A and B, Entinostat treatment induces STAT3 acetylation. HepG2 cells or splenocytes were treated for 6 hours, harvested, and lyzed for Western immunobloting or immunoprecipitation. A, Entinostat induced STAT3 acetylation in HepG2 cells. Upper panel: Cell lysates were analyzed directly and blotted with anti-STAT3 and actin. Bottom panel, Cell lysates were immunoprecipitated with anti-STAT3 antibody and then blotted with anti-STAT3 antibody and with anti-acetylated lysine antibody. B, Entinostat induced STAT3 acetylation in splenocytes. IP and Western blot were performed as described in A. C and D, Down-regulation of Foxp3 is inhibited by blocking STAT3 signaling. Splenocytes were harvested from BALB/c mice and put in culture. Cultures were treated with vehicle or 0.5 mM specific STAT3 peptide inhibitor for one hour, followed by vehicle or 0.5 µM entinostat treatment for 23 hours. Cells were harvested and analyzed by flow cytometry using fluorescence-conjugated antibodies specific to CD4 and Foxp3. Fixable live/dead dye was used to stain cells and live cells were gated. Cell culture was in absence (panel C) or in presence (panel D) of IL-2. In each condition, histograms show quantification of Foxp3 expression in Tregs by FACS analysis. Graph shows means, error bars represent standard deviations. In C, *p* = 0.0002 for entinostat vs. STAT3 inhibitor+entinostat. In D, *p* = 0.00015 for entinostat vs. STAT3 inhibitor+entinostat. Results are representative of three separate experiments.

We next examined possible signaling mediators responsible for Foxp3 down-regulation induced by entinostat. STAT3 signaling is activated by acetylation and has been implicated in Foxp3 modulation [37], [38]. To test whether STAT3 is one of the targets of entinostat, HepG2 cells, a hepatoma cell line with inducible STAT3 signaling used for STAT3 signaling studies [38], were treated for 6 hours. Treatment with 0.5 µM entinostat was sufficient to induce acetylation of STAT3 (Fig. 6A) without significantly changing total STAT3 protein levels (Fig. 6A). In addition, we tested STAT3 acetylation in splenocytes. Again, entinostat treatment increased acetylation of STAT3 in splenocytes (Fig. 6B). To further test whether STAT3 is mediating down-regulation of Foxp3 by entinostat, we used a highly specific, cell permeable peptide STAT3 inhibitor [39]. Entinostat treatment reduced Foxp3 levels in Tregs, whereas the presence of the STAT3 specific inhibitor partially, but significantly neutralized the inhibitory effect of entinostat on Foxp3 expression in Tregs (∼50% neutralization) in both the absence and presence of IL-2 (Fig. 6C and 6D, respectively). In all of the conditions, there was no significant difference in the number of Tregs. This result suggests that STAT3 is in part involved in entinostat down-regulation of Foxp3 expression in Tregs.

### Class I, but not Class II HDAC inhibition suppresses Foxp3 expression in Tregs *in vitro*

**Figure 7:**
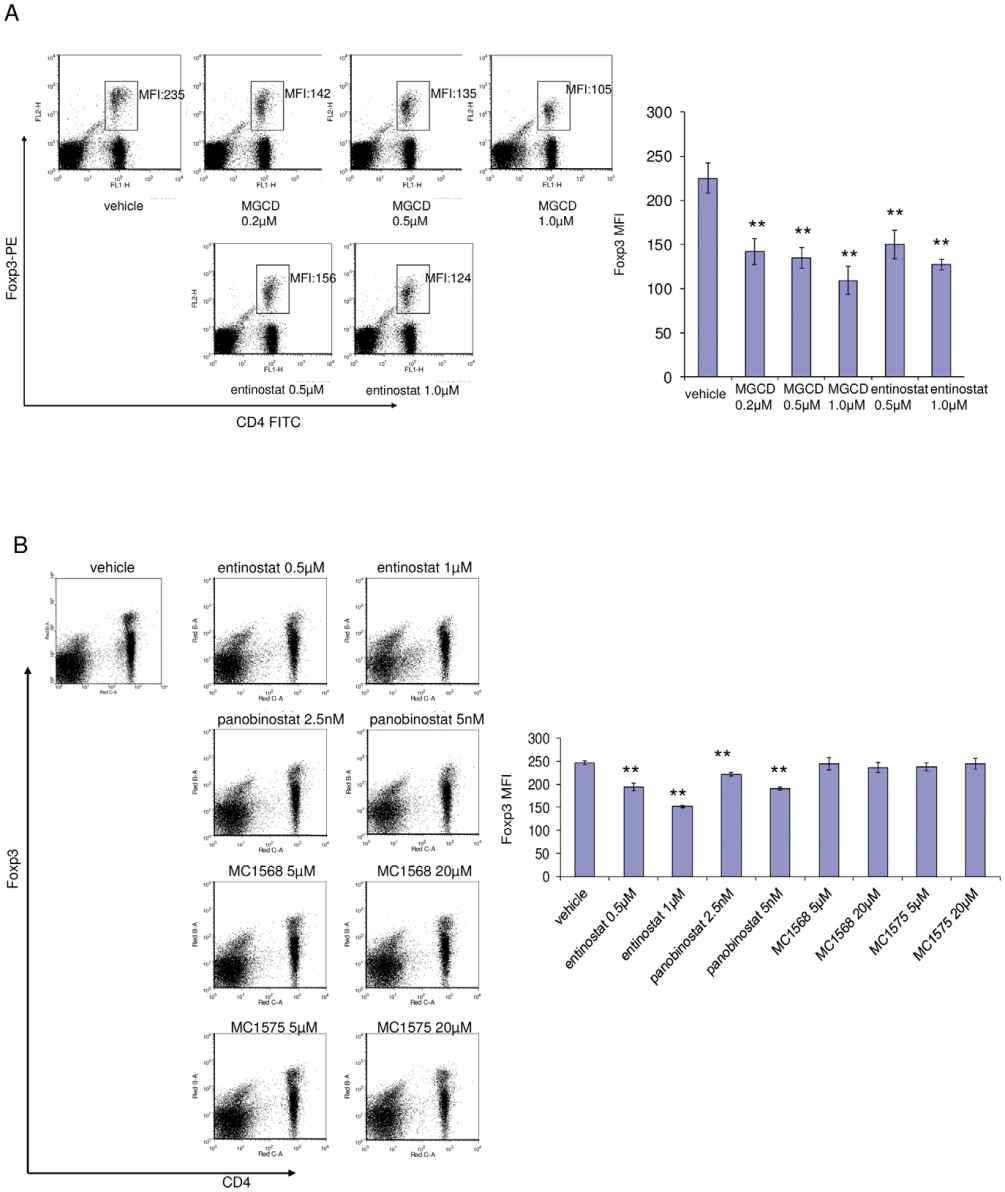
Class I inhibition, not class II inhibition suppresses Foxp3 expression in Tregs. Splenocytes were isolated from BALB/c mice and put in culture with different treatment conditions as indicated for 24 hours. Cells were harvested, stained for surface markers and intracellular Foxp3, and subjected to FACS analysis. Plot gating and parameter indication were described in Figure 1. Histograms show quantification of Foxp3 levels in Tregs. A, Both class I HDAC inhibitors, entinostat and MGCD0103, inhibit Foxp3 expression in Tregs. When comparing entinostat with MGCD0103, PE conjugated anti-Foxp3 antibody was used. B, Class I HDAC inhibitor, entinostat, and a pan inhibitor, panobinostat, but not class II HDAC inhibitors, MC1568 and MC1575, inhibit Foxp3 expression in Tregs. When comparing entinostat with class II HDAC inhibitors and the pan inhibitor, panobinostat, the Alexa 700 conjugated anti-Foxp3 antibody was used since other fluorochrome channels are interfered by the autofluorescence of the class II HDAC inhibitors. ** represents *p*<0.01 for marked treatment vs. vehicle.

Previous studies have reported that inhibition of HDACs increases Tregs number, and promotes Tregs function and associated immune response suppression [30], [40]. Hence, we investigated whether inhibition of different classes of HDACs may have differential effects on Tregs. We tested other HDAC inhibitors including the selective class I inhibitor, MGCD0103, the pan inhibitor, panobinostat, and two selective class II inhibitors, MC1568 and MC1575 [41]. Splenocytes isolated from BALB/c mice were cultured with different treatments for 24 hours. The doses of inhibitors were chosen based on previous studies [41], [42]. Cells were harvested, stained for surface markers and Foxp3, and subjected to FACS analysis. Both class I HDAC inhibitors, entinostat and MGCD0103, down-regulated Foxp3 in Tregs (Fig. 7A). Both entinostat and panobinostat reduced Foxp3 protein levels in Tregs population in a dose-dependent manner. Selective Class II HDAC inhibitors did not have a significant effect on Tregs (Fig. 7B). These results suggest that inhibition of class I, not class II HDACs leads to down-regulation of Foxp3.

## Discussion

In our study, we provide evidence that the class I HDAC inhibitor, entinostat, inhibits Tregs and enhances the antitumor effect of two different immunotherapies. In the entinostat and IL-2 combination strategy, IL-2 treatment activated and promoted proliferation of Teffs, but also activated Tregs (Fig. 1). Low dose entinostat, in combination with IL-2, did not have a direct cytotoxicity against tumor cells. In contrast, entinostat targeted Tregs activity, while IL-2 activated Teffs, with consequent enhancement of the antitumor immune response. Entinostat reduced IL-2 induced elevated Foxp3 levels and counteracted the Treg-promoting “side effect” of IL-2 treatment. This opposite action of entinostat and IL-2 on Tregs may be responsible in part for the *in vivo* synergistic antitumor activity observed with this combination. In the SurVaxM and entinostat combination strategy, the peptide vaccine treatment aimed at inducing antigen-specific immune response, while the entinostat targeted Tregs as part of immunosuppressive environment in tumor-bearing animals. By counteracting the Treg function, entinostat likely allowed for the generation of antigen specific Teffs and facilitated the activation of T effectors to kill target tumors cells. Antigen specific CD8 cells were induced by both vaccine single and combination treatments, but only combination treatment led to enhanced CD8^+^IFN-γ^+^ cells induction in this model (Fig. 4). This result suggests that entinostat may facilitate the activation of antigen specific CD8^+^ T cells through additional CD4^+^ T cell helper support. This is the first study, to our knowledge, to show that the class I HDAC inhibitor, entinostat, in combination with a vaccine therapy, enhances prostate tumor response. The results from these two strategies demonstrate that the application of entinostat may be general and versatile to support different antitumor immunotherapies.

Several previous findings from our group also support the notion that the effect of entinostat in combination with immunotherapy results from immunomodulatory activity rather than a direct cytotoxic effect against tumor cells [25]. First, the combination strategy does not have a synergistic effect in immunodeficient mice. Secondly, survival benefit from the combination therapy was abrogated by depletion of CD8 T cells in immunocompetent mice [25]. In addition, we used a suboptimal dose of entinostat, 5 mg/kg (optimal dose is more than 20 mg/kg in order to direct target tumor cells). The median plasma concentration 20.6±5.01 ng/ml achieved from this dose had no or minimal direct antitumor cytotoxic effect *in vitro* [25]. However, this dose appears to modulate immune response. A higher dose (20 mg/kg) of entinostat did not have the synergistic antitumor effect observed with a lower dose possibly due to toxicity to Teffs (Fig. 5).

Previous reports have suggested that HDAC inhibition leads to reduced immune response by promoting Tregs and down-regulating pro-inflammatory cytokines [26], [30]. A recent study has shown the class I/II HDAC inhibitor TSA promoted Foxp3 expression and the generation and function of Tregs in an autoimmune disease murine model with C57BL/6 mice [30]. Under our experimental conditions utilizing BALB/c mice and the RENCA tumor model, TSA (1 mg/kg/day) did not induce changes in either number or Foxp3 expression of Tregs (data not shown). The strain difference may play a role in these different observations since BALB/c mice have increased number of Tregs and show a different response to suppression of their Teffs as compared to C57BL/6 mice [43]. In addition, TSA has different pharmacokinetic features from other HDAC inhibitors [44]. TSA undergoes rapid and extensive metabolism once absorbed and is rapidly inactivated in rodents. It has been suggested that inhibition of class II HDAC9 by TSA induces acetylation of Foxp3 protein, which enhances Treg suppression function [30]. TSA also inhibits class I HDACs, but at the dose used in the study, a class II HDAC inhibition induced effect on Tregs may be dominant. Inhibition of the class III HDAC, SIRT1, also induced acetylation-mediated Foxp3 protein stabilization, which led to an increase of Tregs functionality [45]. In contrast, our study demonstrates an opposite effect on Tregs by inhibition of class I HDACs. Both low and high doses of the class I HDAC inhibitor, entinostat, suppressed the inhibitory effect of Tregs (Fig. 5). Taken together, these results indicate that class I HDAC inhibition and class II inhibition may have a different or even an opposite effect on Tregs. Additional comparisons between different types of HDAC inhibitors suggest only class I HDAC inhibition down-regulated Foxp3 (Fig. 7). Inhibition of class II HDACs may promote Tregs function through different mechanisms of action [30]. These considerations have direct clinical impacts in designing rational combination clinical trials with HDAC inhibitors and immunotherapies.

Increased levels of Tregs, or increased expression of Foxp3 and enhanced Tregs function have been reported in cancer patients, including kidney and prostate cancer patients [2], [8], [9]. IL-2 induces Tregs expansion in normal individuals, and more extensively in lymphopenic cancer patients [10], which may impair its antitumor immune response. Several studies have shown that Tregs reduce the efficacy of immunotherapy [15] and depletion of Tregs enhances antitumor immune responses [9]. Tregs are also induced in cancer patients receiving high dose IL-2 [11], [46]. However, a decrease in Tregs has been associated with objective clinical response to IL-2 therapy [11]. The mechanism responsible for these observations has not previously been elucidated, but these clinical reports suggest that depletion of Tregs may enhance the ability of IL-2 to elicit an antitumor immune response in cancer patients. Some studies have also suggested that Tregs are an important immunosuppressive component that leads to irresponsiveness to and limited efficacy of vaccine therapy. The Tregs depletion reagents in development are anti-CD25 antibody or toxin conjugated IL-2. These reagents target cells with the CD25 surface marker. The depletion effect of these reagents may not be specific to Tregs since activated T effectors also express the CD25 surface marker while Tregs stably express CD25. Entinostat treatment appears to have an advantage over current approaches as it selectively inhibited Tregs by down-regulating Foxp3 expression without affecting Teffs proliferation (Fig. 5). No significant differences were observed in the level of CD8^+^, natural killer, and natural killer T cells after entinostat treatment [25].

Foxp3 protein is essential for the development and function of Tregs. Other cell types such as CD4^+^CD25^−^ T cells can acquire immunosuppressive activity if induced to express Foxp3 [47], suggesting that Foxp3 expression is sufficient to drive the suppressive function. In general, histone hyperacetylation at certain loci induces gene expression. Negative regulation of Foxp3 by entinostat treatment is unlikely related to histone acetylation at the Foxp3 site, but rather to the modulation of upstream pathways. Our study suggests a class I HDAC substrate protein (non-histone) is modified by entinostat treatment and is responsible for its Foxp3 transcriptional regulation. Down-regulation of Foxp3 by IL-6 and IL-27 has been reported to be STAT3-dependent [48], [49]. STAT3 protein is activated by acetylation [37], [38] via CBP/p300 *in vivo*, and interacts with class I HDACs (HDAC 3 displayed strongest interaction) [37]. It is conceivable that inhibition of class I HDACs by entinostat induces STAT3 acetylation and facilitates its signaling with consequent down-regulation of Foxp3. Our results also show that blockage of the STAT3 pathway partially inhibits the down-regulation of Foxp3 by entinostat (Fig. 6), and suggests that STAT3 is at least in part responsible for this effect. Blockage of STAT3 by the specific peptide inhibitor might not have been optimal in our setting because we were unable to reach the recommended concentration [39]. Interestingly, one of the transcriptional partners of Foxp3 in Tregs, Runx, controls Foxp3 expression by interacting with CBFβ [50]. Additional mechanisms responsible for the regulation of Foxp3 expression by HDAC inhibitors are now under investigation in our group. This will not only provide additional evidence supporting the utilization of these agents in combination with immunotherapy strategies, but will also identify new targets for therapeutic interventions.

In summary, our study suggests a novel mechanism of the *in vivo* antitumor effect of HDAC inhibitors. Entinostat has an immunomodulatory ability by inhibiting Tregs and consequently enhancing an IL-2 and vaccine induced antitumor response. This combination strategy also has promising potential to be effective in other immunotherapies and in different tumors. A clinical trial of combinational therapy with high dose IL-2 and entinostat in metastatic renal cell carcinoma patients has been initiated at our institution.

## Materials and Methods

### Cells

The murine renal cell carcinoma cell line RENCA was purchased from American Type Culture Collection (National Cancer Institute) and cultured in RPMI 1640 (Life Technologies) with 10% fetal bovine serum (Sigma-Aldrich) and 1% Pen/Strep (Life Technologies, Invitrogen, Gibco), and incubated at 37°C in an atmosphere containing 5% CO_2_. Myc-CaP cell line was cultured in DMEM (Mediatech, Inc.) with 10% FBS. For isolation of splenocytes, five- to six-week-old female BALB/c mouse (National Cancer Institute) spleens were harvested, mashed on, and passed through a 70 µm strainer. These cell suspensions were centrifuged at 300 g for 10 min at 4°C. Cell pellets were treated with ACK lysing buffer (Biosource). Splenocytes were then resuspended and cultured in complete media (RPMI supplemented with 10% FBS, 1 mM sodium pyruvate, 100 µM non-essential amino acid, 2 mM L-glutamine, Pen (100 units/ml)-Strep (100 µg/ml) and 55 µM β-mecaptoethanol). For *in vitro* treatments, cells were incubated in media with entinostat (kindly provided by Syndax Pharmaceuticals, Inc.), MGCD0103 (Active Biochemicals Co. Ltd.), MC1575 and MC1568 (kindly provided by Dr. Antonello Mai at Università degli Studi di Roma “La Sapienza”, Rome, Italy), panobinostat (Novartis) or vehicle (0.1% DMSO in the media) with or without supplement of 20 U/ml of IL-2 (Invitrogen).

### Isolation of regulatory T cells

Spleens and lymph nodes were harvested and cell suspensions were made as described above. Tregs (CD4^+^ CD25^+^ cells) and T effector cells (Teffs, CD4^+^ CD25^−^ cells) were enriched using a Treg isolation kit according to the manufacturer's instructions (Miltenyi Biotech).

### RNA analysis

Total cellular RNA was isolated with RNeasy mini kit (Qiagen). cDNAs were synthesized with superscript reverse transcriptase (Invitrogen). Quantitative PCR was performed with icycler (Bio-Rad) or ABI 7300 (Applied Biosystem). Primers for Foxp3 cDNA: Forward: 5′-TTA TCC AGC CTG CCT CTG AC-3′; Reverse: 5′-AGC CCC TGG TCC CTA GAA GT-3′. cDNA inputs were normalized to housekeeping gene GAPDH RNA or ribosomal RNA RPL13A. Primers for reference gene GAPDH: Forward: 5′-AAT GTA TCC GTT GTG GAT CTG A-3′; Reverse: 5′- GCC TGC TTC ACC ACC TTC T-3′. RPL13A: Forward: 5′-GAG GTC GGG TGG AAG TAC CA-3′; Reverse: 5′-TGC ATC TTG GCC TTT TCC TT-3′.

### Immunoprecipitation and Western blot analysis

HepG2 cells were treated with vehicle, 0.5 µM, or 2 µM entinostat for 6 hours before harvest. Cell pellets were lysed in non-denaturing lysis buffer (20 mM Tris HCl, pH8, 137 mM NaCL, 10% glycerol, 1% Nonidet P-40 (NP-40), 2 mM EDTA). The cell lysates were immunoprecipitated with anti-STAT3 (C-20) (Santa Cruz Biotechnology) and protein G Dyna® beads (Invitrogen). The beads were washed in lysis buffer, eluted by re-suspension in loading buffer, and boiled for 5 minutes. The samples were analyzed by Western blot with anti-acetylated lysine (Cell Signaling), anti-STAT3 antibodies (Santa Cruz) or anti-Foxp3 (eBioscience). The details of Western blot analyses have been previously described [51].

### Immunohistochemistry

Tumor pieces were fixed in 10% formalin and embedded in paraffin blocks. 4 µm sections were stained according to detailed methods described previously [25]. Rat anti-mouse/rat Foxp3 antibody (eBioscience) was used to stain Tregs. Rat IgG was used as a negative control.

### Regulatory T cell suppressive functional assay

Isolated Teffs (CD4^+^CD25^−^) were labeled with carboxyfluorescein diacetate succinimidyl ester (CFDA-SE or CFSE) and cultured in complete medium with stimulations, including anti-mouse CD3ε antibody (0.5 µg/ml) and antigen presenting cells (CD4^+^ cells-depleted, irradiated splenocytes). Tregs were added to the culture in different ratios to Teffs. After a 60–72 hour incubation, all cells in culture were harvested and stained for CD4-APC. Dividing cells were analyzed by calculating percentage of cells with diluted CFDA-SE compared to the original undivided Teff population. Cell events were acquired using FACSCalibur and CellQuest. Data were analyzed with FCS Express (De Novo Software).

### In vivo tumor growth

The animal protocol was approved by the Institutional Animal Care and Use Committee at Roswell Park Cancer Institute (protocol 1137 M), and was in accordance with the NIH Guide for the Care and Use of Laboratory Animals. Five- to six-week-old female BALB/c mice (National Cancer Institute) were kept in a temperature-controlled room on a 12/12 hour light/dark schedule with food and water *ad libitum*. RENCA cells (5×10^5^) harvested from non-confluent monolayer cell cultures in 20 µL of 1× HBSS were injected under the renal capsule. Animals were randomly distributed into four groups (5–7 animals/group): vehicle (0.5% methocel in saline, daily, 7 d/wk by oral gavage), IL-2 (Prometheus, 150,000 IU, twice a day, 2 d/wk, i.p.), entinostat (5 mg/kg, daily, 5 d/wk by oral gavage), combination of IL-2 and entinostat. The animals were treated for two to three weeks and then euthanized by carbon dioxide inhalation. At the end of the experiment, tumors and spleens were collected. The weight of the healthy right kidney was subtracted from the RENCA-injected kidney.

Castration resistant tumor [35] was developed from Myc-CaP cell lines derived from the Hi-Myc transgenic prostate cancer mouse model [36]. Small pieces of the tumor (1 mm^3^) were inoculated subcutaneously in the right flank of castrated male FVB mice. Animals were randomly distributed into four treatment groups (7–9 animals/group): vehicle, vaccine (SurVaxM), entinostat, or combination. SurVaxM is a survivin peptide vaccine composed of 15 amino acids with one amino acid alteration from wild type sequence [33]. Mice were given 100 µg of SurVaxM peptide and 100 ng of GM-CSF by subcutaneous injection, once per week. At the end of the 3–4 week experiment, tumors and spleens were collected and subjected to analysis.

### Cell staining and flow cytometry

Splenocytes, lymph node cells or peripheral blood cells were washed with flow buffer which included PBS with 1% of FBS and 2 mmol/L of EDTA, then blocked with 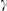 III/II R Ab (BD Pharmingen) and stained with antibody against surface markers such as CD4-FITC, CD4-APC, CD25-APC, and CD8-FITC (BD Pharmingen). Cells were then fixed in Fix/Perm buffer (eBioscience) and stained with antibodies against intercellular proteins such as anti-mouse Foxp3 antibody (FJK-16, eBioscience). Cells stained with specific antibodies, as well as isotype control stained cells, were assayed on a FACScalibur (BD Biosciences) or a LSR II flow cytometer (BD Biosciences). Data analysis was performed using FCS Express software.

### IFN-γ induction assay

1×10^6^ splenocytes from mice that received different treatments were cultured with stimulation of PMA (Sigma, 20 ng/ml) and Ionomycin (Sigma, 1 µg/ml) for 5 hours. Brefeldin A (Sigma) was added to the cultures to block the protein secretion. Cells were harvested and stained for surface markers, then fixed and stained for intracellular IFN-*γ* (eBioscience).

### Antigen specific tetramer binding assay

Splenocytes (1×10^6^) were incubated with 10 ml of iTAg MHC Class I Murine H2-K^b^ Tetramer-SA-PE bound by MFFCFKEL peptide with specificity for SurVaxM (Beckman Coulter) or iTAg MHC Class I Murine H2-Kb Tetramer-SA-PE bound by SIINFEKL ovalbumin peptide to represent negative control (Beckman Coulter) for 30 minutes. Samples were also labeled with 10 ml anti-CD8-FITC (clone 53.6.7; BioLegend). Following incubation, 1 ml of iTAg MHC Tetramer Lyse Reagent (Beckman Coulter) supplemented with 25 ml iTAg MHC Tetramer Fix Reagent (Beckman Coulter) was added to the samples, which were then incubated for 10 minutes at room temperature, subsequently washed with PBS, and resuspended in 400 ml of FluoroFix Buffer (BioLegend).

### Statistical analysis

Differences between experimental groups were tested by either Student's t test or for variances by ANOVA. *p*<0.05 was considered statistically significant.

## Supporting Information

Figure S1 **Tumor infiltration of Tregs.** Tumor sections from differently treated mice were stained with anti-mouse/rat Foxp3 antibody. Representative images with 20× resolution are showed. Arrow in each image points one of the stained Tregs.
(TIF)
