# Supplementary figures and images for: Class I Histone Deacetylase Inhibitor Entinostat Suppresses Regulatory T Cells and Enhances Immunotherapies in Renal and Prostate Cancer Models
Source: PLoS One. 2012 Jan 27;7(1):e30815. doi: 10.1371/journal.pone.0030815 (PMC3267747; doi:10.1371/journal.pone.0030815)

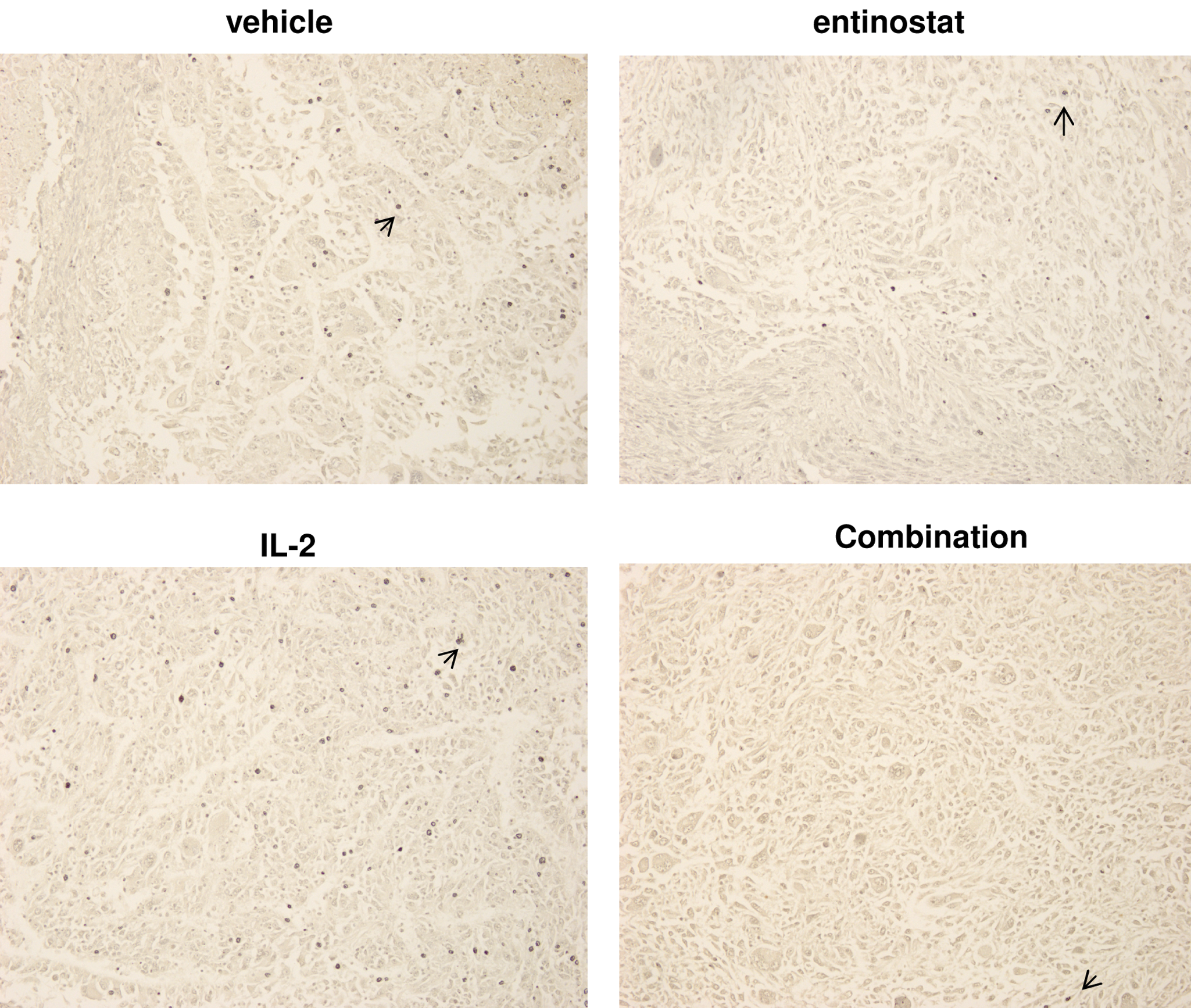

Supplement: Figure S1 — Tumor infiltration of Tregs. Tumor sections from differently treated mice were stained with anti-mouse/rat Foxp3 antibody. Representative images with 20× resolution are showed. Arrow in each image points one of the stained Tregs. (TIF) [file pone.0030815.s001.tif]
